# Recombinant Human CD19 in CHO-K1 Cells: Glycosylation Patterns as a Quality Attribute of High Yield Processes

**DOI:** 10.3390/ijms241310891

**Published:** 2023-06-30

**Authors:** Magdalena Billerhart, Monika Hunjadi, Vanessa Hawlin, Clemens Grünwald-Gruber, Daniel Maresch, Patrick Mayrhofer, Renate Kunert

**Affiliations:** 1 Institute of Animal Cell Technology and Systems Biology, Department of Biotechnology, University of Natural Resources and Life Sciences, Muthgasse 18, 1190 Vienna, Austria; magdalena.billerhart@boku.ac.at (M.B.); monika.hunjadi@boku.ac.at (M.H.);; 2 BOKU Core Facility Mass Spectrometry, University of Natural Resources and Life Sciences, Muthgasse 11, 1190 Vienna, Austria; clemens.gruber@boku.ac.at (C.G.-G.);

**Keywords:** recombinant CD19 glycosylation pattern, homeostasis in high cell density cultures, CD19-AD2 fusion protein, hypothermic cultivation, recombinant CD19 for CAR-T cell evaluation

## Abstract

CD19 is an essential protein in personalized CD19-targeting chimeric antigen receptor (CAR)-T cell-based cancer immunotherapies and CAR-T cell functionality evaluation. However, the recombinant expression of this “difficult to-express” (DTE) protein is challenging, and therefore, commercial access to the protein is limited. We have previously described the successful stable expression of our soluble CD19-AD2 fusion protein of the CD19 extracellular part fused with human serum albumin domain 2 (AD2) in CHO-K1 cells. The function, stability, and secretion rate of DTE proteins can be improved by culture conditions, such as reduced temperature and a shorter residence time. Moreover, glycosylation, as one of the most important post-translational modifications, represents a critical quality attribute potentially affecting CAR-T cell effector function and thus impacting therapy’s success. In this study, we increased the production rate of CD19-AD2 by 3.5-fold through applying hypothermic culture conditions. We efficiently improved the purification of our his-tagged CD19-AD2 fusion protein via a Ni-NTA-based affinity column using a stepwise increase in the imidazole concentration. The binding affinity to commercially available anti-CD19 antibodies was evaluated via Bio-Layer Interferometry (BLI). Furthermore, we revealed glycosylation patterns via Electrospray Ionization Mass Spectrometry (ESI–MS), and five highly sialylated and multi-antennary N-glycosylation sites were identified. In summary, we optimized the CD19-AD2 production and purification process and were the first to characterize five highly complex N-glycosylation sites.

## 1. Introduction

Highly complex or artificial proteins, such as bispecific molecules or mutants from native proteins, are becoming more and more recognized for diagnostics and therapy [1,2,3,4,5], and, therefore, optimized expression platforms need to be developed.

Difficult-to-express (DTE) proteins are often underrepresented in scientific and technological publications, since monoclonal antibodies (mAb) have become the dominant class in the biopharmaceutical market [1,6,7,8,9], possessing high expression levels [10], relatively simple glycosylation with only one N-glycosylation site that is practically non-sialylated [11], and established analysis and purification methods [12].

We previously described the successful generation of a CHO-K1 cell line expressing a novel DTE CD19-AD2 fusion construct consisting of the extracellular domain (ECD) of native human CD19 fused to domain two of the human serum albumin (CD19-AD2) molecule (Supplementary Figure S1). This soluble CD19-AD2 fusion protein can be used for the functional evaluation of personalized CD19-targeted chimeric antigen receptor (CAR)-T cell-based cancer immunotherapies. We already demonstrated the binding and biological activity of our CD19-AD2 fusion construct by flow cytometric analysis and effective stimulation of CD19-CAR-T cells using CD19-AD2-decorated planar-supported lipid bilayers, respectively [13]. Moreover, the successful binding of CD19-AD2 to patients’ primary human CAR T cells was confirmed [2].

Careful selection of bioprocess conditions guarantees that the protein can be brought downstream in high quantity and at the desired quality [14,15,16]. Mild hypothermic culture conditions lead to a reduced growth rate, increased culture longevity, and cell-specific productivity (qP) in CHO cells, expressing a wide range of recombinant proteins [14,17,18,19,20]. Further, a temperature downshift has been reported to reduce high molecular weight protein species, resulting in lower acidic and higher basic charge variants, as well as elevated levels of N-glycans [21,22]. With a reduced residence time in the bioreactor combined with a lower temperature, the most efficient maturation of the proteins, and the associated increase in secretion, can be enabled [15,16,23].

Here, we describe the improvement of recombinant CD19-AD2 expression by hypothermic condition (32 °C vs. 37 °C) in a semi-continuous perfusion bioprocess based on shake tubes, and we investigate the N-glycosylation pattern of the protein, as it is essential for the stability and the function of the mature protein. Recently, Teplyakov et al. were able to crystalize the extracellular domain of a CD19 mutant expressed in insect cells and described that only a CD19 variant depleted in one glycosylation site (N138Q correspondent to our CD19-AD2 site N119 displayed in Supplementary Figure S1) was able to be expressed in moderate amounts [24]. Teplyakov et al. identified N67 and N106 as glycosylated. However, the glycosylation sites N162 and N246, which are in either entirely or partially disordered loops, could not be determined.

Our results here are the first to describe the CD19 N-glycosylation pattern for all five N-glycosylation sites expressed in the mammalian expression host, CHO-K1. The 3.5-fold improved titer and complex N-glycosylation patterns demonstrate an efficient bioprocess workflow under hypothermic conditions, resulting in a stable and functional CD19-AD2 construct with all N-glycosylation sites being occupied and highly sialylated.

## 2. Results

### 2.1. Bioprocess of the CD19-AD2 Fusion Protein at Hypothermic vs. Standard Culture Conditions

**Figure 1:**
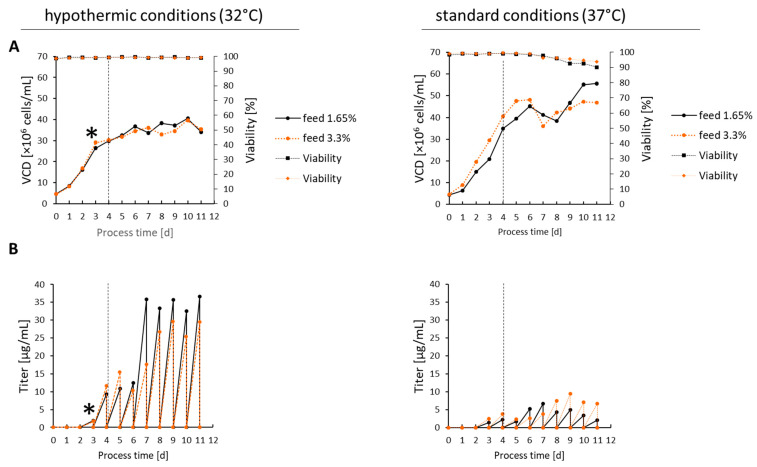
The semi-continuous perfusion cultivation process at 37 °C (right panel) vs. 32 °C (left panel). (**A**) VCD and viability of all process days. (**B**) The volumetric titer of CD19-AD2 determined from the daily harvested supernatant is displayed. The asterisk indicates the temperature shift from 37 °C to 32 °C, and a vertical dashed line indicates the start of feed supplementation on day four.

CD19-AD2 protein expression was performed in a semi-continuous perfusion mode enabled by daily medium exchange. The growth curve, viability, and product formation for two different temperatures and two different feed concentrations are shown in Figure 1. The temperature shift at day three induces a controlled stagnation in cell growth by reaching a viable cell density (VCD) of 40 × 10^6^ cells/mL compared to a maximum of 56 × 10^6^ cells/mL when cultured at 37 °C. Culture conditions at 32 °C benefit higher cell viability compared to those at 37 °C with slightly decreasing viability and dropping VCD on day seven (Figure 1A). Supplementing the medium with different feed concentrations was always started on day four. Hypothermic process conditions improved the CD19-AD2 secretion by 3.5-fold, with a maximal volumetric titer of up to 36.6 µg/mL at 32 °C compared to 9.4 µg/mL at 37 °C in combination with a significantly higher qP (Figure 1B and Supplementary Figure S2). Consequently, the higher amount of CD19-AD2 secretion in 32 °C cultures can be attributed to a reduced growth rate, prevention of nutrient and energy limitation, and the channeling of metabolic energy towards recombinant protein production. Metabolite analyses (Supplementary Figure S3) showed that cultivation at 37 °C resulted in total daily glucose consumption, starting on day four. Cells with less feed supplementation began to take up lactate from the sixth day to compensate for the glucose deficiency. The metabolism between glutamine (Q) and glutamic acid (E) is more complicated, with much room for interpretation due to the glutamine synthetase endogenously present in CHO-K1 cells. At 37 °C, we observed an almost complete consumption of Q and E accompanied by a loss in viability and VCD on day seven. In contrast, at 32 °C, we saw a remaining reservoir of Q every day, and accordingly, the secreted amount of ammonium was lower than at 37 °C.

### 2.2. Purification of CD19-AD2

**Figure 2:**
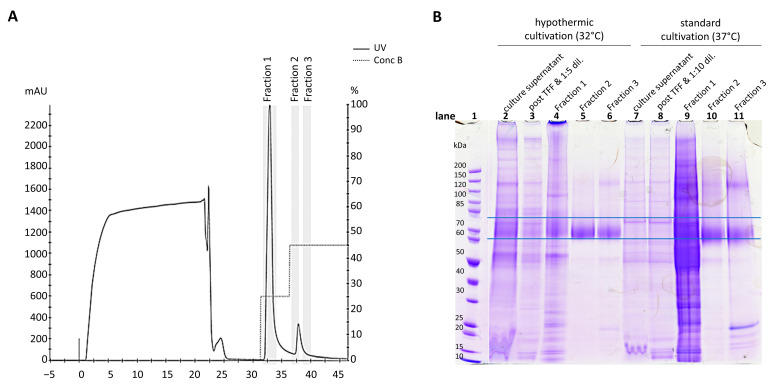
CD19-AD2 protein purification. (**A**) The HisTrap profile example for the stepwise elution of CD19-AD2 fusion protein with 25% and 45% of 500 mM imidazole, containing elution buffer (pH 7.4). The grey highlighter indicates the peak fractions of host cell proteins (fraction 1) and CD19-AD2 (fractions 2 and 3). (**B**) The quality of the purified protein was verified by non-reducing SDS-PAGE/Coomassie staining. Equivolumetric amounts of the pooled culture supernatant, 1:5 diluted (32 °C) or 1:10 diluted (37°) concentrated and rebuffered supernatant, as well as elution fractions 1 to 3, were loaded. The area between the horizontal lines represents the highly glycosylated CD19-AD2.

Concentrated and rebuffered culture supernatants (40 mL from 32 °C, 5× concentrated or 107 mL from 37 °C, 10× concentrated) were loaded onto the HisTrap column, and proteins were separated with a stepwise imidazole gradient. A representative profile for CD19-AD2 purification is displayed in Figure 2A. The stepwise elution separated host cell proteins in the 25% imidazole concentration fraction (Figure 2A, fraction 1), followed by the his-tagged CD19-AD2 fractions, indicated by a single peak after applying 45% imidazole (Figure 2A, fractions 2 and 3). The pooled culture supernatants (Figure 2B, lanes 2 and 7), tangential flow filtration (TFF) concentrated and rebuffered samples (lanes 3 and 8), and elution fractions (lanes 4–6 and 9–11) were analyzed via SDS-PAGE (Figure 2B). In lanes 4 and 9, the 25% imidazole fraction 1 eluted mainly host cell proteins. The more intense band in lane 9, from cells cultured at 37 °C, is due to the higher protein amount caused by the higher volume of supernatant used. The 45% imidazole elution fractions were analyzed, resulting in a prominent band of purified CD19-AD2 protein at 60–80 kDa (Figure 2B, lanes 5, 6, and 10, 11, respectively). Fraction 2 of the 32 °C and the 37 °C cultivations was used for further glycosylation analysis.

### 2.3. The Binding Kinetics of CD19-AD2 to Different Commercially Available mAbs

Real-time measurement of association (k_on_) and dissociation rates (k_off_) of purified CD19-AD2 to various commercially available anti-CD19 antibodies are the basis for the calculation of their equilibrium dissociation constants K_D_s, which are the ratios of k_off_/k_on_ (Table 1 is sorted by decreasing k_on_). The lower the K_D_, the tighter the binding of the analyte CD19-AD2 to the antibody ligand and the more stable the interaction. Among the tested antibodies, FMC63 showed the lowest K_D_ value with 3.19 nM and thus the highest binding affinity to CD19-AD2 compared to 3B10 with 19.12 nM or HIB19 with a K_D_ of 38.29 (Table 1). Remarkably, CD19-AD2 did not bind to 4G7 in this setup.

**Figure 3:**
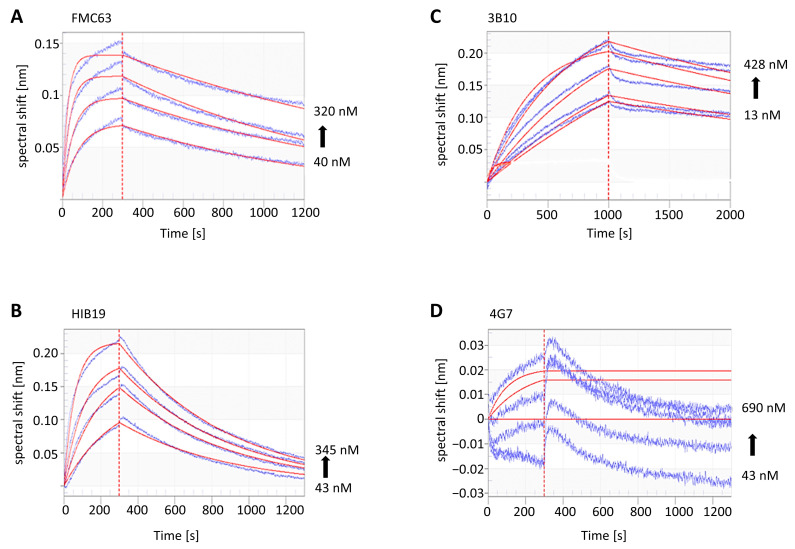
Real-time binding sensorgrams of the immobilized biotinylated anti-CD19 antibodies (ligand concentration 10 µg/mL) and CD19-AD2 construct (analyte). (**A**) FMC63-btn; (**B**) HIB19-btn; (**C**) 3B10-btn; (**D**) 4G7-btn were captured on SA biosensors and dipped in wells containing the analyte CD19-AD2 at different concentrations between 10 and 600 nM. The spectral shift, corresponding to the thickness of the biolayer, differs for individual analyses. The binding signals (blue sensorgrams) were obtained by subtracting the signals from ligand-coated SA biosensors dipped in wells with buffer (non-specific binding control). Fitted curves are depicted as red lines and were obtained by global fitting using a 1:1 ligand model.

The corresponding kinetic binding sensorgrams are visualized in Figure 3, displaying FMC63 with the highest k_on_, indicated by a steep association curve and an intermediate k_off_ with a slightly declining dissociation curve compared to the other antibodies tested. Thus, FMC63 has the highest affinity for CD19-AD2, explained by the fastest binding and an intermediate dissociation time among the three antibodies. In the case of HIB19, the analyte CD19-AD2 binds slower indicated by a flatter binding curve for k_on_, while the dissociation curve is steeper, and thus CD19-AD2 is released faster, thus represented by a high k_off_.

### 2.4. CD19-AD2 Glycoprofile Revealing Highly Complex Glycans

The glycoprofile of our CD19-AD2 construct, produced under standard or hypothermic conditions, was analyzed, and all five potential N-glycosylation sites were identified.

**Figure 4:**
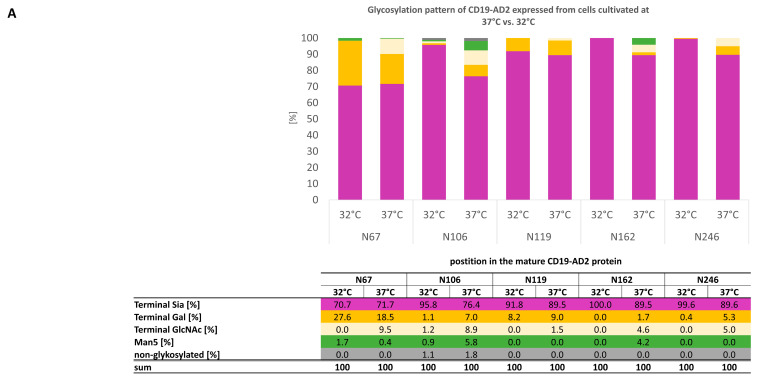

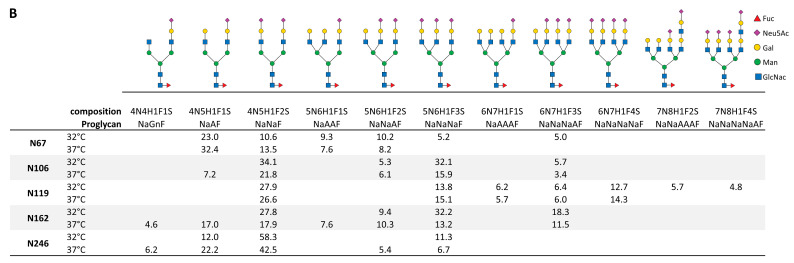
Glycosylation pattern of CD19-AD2 expressed in CHO-K1 cells cultivated at 37 °C vs. 32 °C. The five N-glycosylation sites at position N67, N106, N119, N162, and N246 are characterized. (**A**) Color-coded bar graph of the site-specific glycosylation as relative proportions of found glycoforms with a table legend. (**B**) Representative structures of sialylated N-glycans found and their percentual representation are displayed extra in table format (threshold of prevalence > 4.5%).

We clustered the glycoforms according to the sites on the protein (N67, N106, N119, N162, N246) and for the complexity of the individual glycans represented as relative proportions in % of found glycoforms (Figure 4A). All glycosylation sites show a high percentage of 70–100% sialic acid (indicated as “Terminal Sia”), although, in Figure 4A, we did not distinguish whether one or more molecules of sialic acid are bound per glycan. Complex glycans without sialic acid are designated as “Terminal Gal”, indicating at least one galactose at the glycan. The sum of sialylated and terminal galactose-carrying glycans on each glycosylation site is above 96% for the CD19-AD2 generated at 32 °C and above 83% when cultivated at 37 °C. Almost no unglycosylated sites were detected, so missing highly complex glycans were mostly found as glycans with “terminal GlcNAc”. In general, a low amount of mannose glycans were detected, with the highest one on asparagine N106 (5.8%) and N162 (4.2%) in the 37 °C-derived CD19-AD2. Only N67 showed a slightly lower amount of high mannose glycans at 37 °C (0.4%), as opposed to 32 °C (1.7%).

Having a deeper look at the distribution of sialylated glycans among glycosylation sites and cultivation temperatures, we identified higher sialylation when CD19-AD2 was cultivated at 32 °C (Figure 4A), with the exception of N67, showing similar sialylation at both temperatures. Highly complex, sialylated glycans with bi- to tetra-antennary structures and also unusual oligo-glycans carrying more than four galactose molecules per glycan, were identified. The associated structures of all identified sialo-glycans shown in Figure 4B, without having performed a steric structure analysis, are representatives that can arise in different isomeric compositions. Furthermore, all sialylated glycans were found to be fucosylated. Glycan forms with more than six N-Acetylglucosamines and more than four galactoses are indicated as lactosamin-repeats (LacNAc-repeats) [25,26,27,28], which were exclusively detected at position N119 and slightly more present at cells cultured at 32 °C. The corresponding mass spectra (Supplementary Figure S4) and a complete list of all found glycans (Supplementary Table S1), including glycans with N-glycolylneuraminic acid (Neu5Gc), which showed a prevalence <4.5%, are represented in the supplements.

The glycosylation of the native protein produced at 32 °C vs. 37 °C conditions was confirmed via silver staining by SDS-PAGE of the original vs. the PNGase F-deglycosylated CD19-AD2. Under non-reducing and DTT reducing conditions, similar bands at both temperatures were observed. These data are in accordance with our data, which are already published in [13].

## 3. Discussion

Our CD19-AD2 fusion protein was stably expressed in recombinant CHO-K1 cells, and, as described for some other proteins [19,20], the production rate was significantly increased when producing the protein under hypothermic conditions. The highest productivity was accomplished by a cultivation process starting with a growth phase at 37 °C until reaching a VCD of more than 20 × 10^6^ cells/mL, followed by a production phase at 32 °C with daily medium exchange to keep the cells in physiological homeostasis and to reduce the residence time of the expressed protein [15,16]. Under hypothermic conditions, a 3.5-time higher CD19-AD2 yield could be generated (Figure 1B). Considering the specific production rate, the hypothermic process averages even up to five times more, with 0.5 pg/cell/day compared to recombinant protein production of 0.1 pg/cell/day under standard conditions at 37 °C due to a lower VCD, but a higher titer (Supplementary Figure S2). During cultivation at 37 °C, nutrient limitation occurs, and, therefore, higher feed leads to higher qP. At 32 °C, nutrient limitation can be largely prevented, so more feed does not lead to an improvement in product production, on the contrary.

Our improved purification protocol with a stepwise imidazole elution was more efficient at receiving a precise separation of the host cell protein at the 25% imidazole elution and a highly pure CD19-AD2 product eluted at the 45% imidazole elution step. With this stepwise elution procedure, we were able to purify several mg of pure CD19-AD2 with a single-step purification, starting with 250–1000 mL culture broth.

To the best of our knowledge, we characterized, for the first time, the site-specific N-glycosylation pattern of human CD19. Glycans are essential for the stability and function of secretory and membrane proteins [29]. Heard et al. highlighted that CD19-targeted CAR-T cell effectiveness depends on protein glycosylation. They have shown that the disruption or overexpression of signal peptide peptidase-like 9 causes hyper- and hypo-glycosylation of CD19, enabling resistance to CAR-T cell therapy [30].

The glycosylation studies from our purified CD19-AD2 protein identified complex N-glycans, with di- to tetra-antenna structures, with a very high percentage being sialylated and bearing fucose at all five potential N-glycosylation sites (Figure 4). N-glycans are important for stability and protect therapeutic proteins from denaturation. Early research found that tetra sialylated tetra-antennary N-glycans are determinants of stability and in vivo bioactivity of EPO [29,31,32,33]. Moreover, we found almost no unglycosylated site and only low amounts of unprocessed Man5. Additionally, our analysis revealed LacNAc structures characterized by more than four Gal and more than six HexNAc residues. Interestingly, these structures are mainly found on CD19-AD2 cultivated at 32 °C at position N119. In the literature, LacNAc structures are described for membrane proteins of blood cells, with various receptor/ligand interaction functions [34,35].

Additionally, we confirmed glycosylation, which is essential for the proper folding and compaction of glycoproteins, via SDS-PAGE. The visible smear (Figure 2B) on the electrophoretic gel of CD19-AD2 at 60–80 kDa under non-reducing conditions is caused by the protein backbone of 55 kDa and complex sialylated glycans bearing 10–20 hexoses on each of the five N-glycosylation sites, translating into 9 to 18 kDa for the entire five N-glycosylation sites.

Recently, a comprehensive glycosylation reaction network for CHO cells has been derived, including CHO K1 wild-type cells, suggesting, together with our results, CHO cells as fitting hosts for the production of highly glycosylated therapeutic proteins [28].

Huppa and his group demonstrated, in an in vitro experiment, that CAR-T cells need at least 1000 antigens to be adequately stimulated for delivering a killer response. This is a rather high amount compared to native T cells, requiring only one to five viral antigens for fighting viral-infected cells. This helps to explain tumors’ relapses in almost 50% of patients treated with CAR-T cells due to the downregulation of its antigen density [36,37]. This encouraged us to investigate the binding kinetics of four commercially available anti-CD19 antibodies (FMC63, HIB19, 3B10, and 4G7), including FMC63, whose single-chain variable fragment (scFvs) is the most abundantly used binding sequence in most anti-CD19 CAR-T cells, inclusive of those used for approved CAR-T cell therapy [38,39]. From all the anti-CD19 antibodies tested in our study, FMC63 exhibited the highest binding affinity (K_D_ = 3.19 nM) to CD19-AD2, followed by 3B10 (K_D_ = 19.12 nM), HIB19 (K_D_ = 38.29 nM), and 4G7 (no binding). Due to intensive speculations about the reason for the downregulation of CD19 on tumor cells, an additional reason might be the low dissociation of FMC63 from CD19, causing a clonal selection by immune pressure of CD19-down-regulated tumor cells. Recently, CAR-T cells, based on the high-affinity CD19 binding domain of FMC63, were shown to induce antigen loss due to trogocytosis, stripping CD19 from lymphoma cells and incorporating it into their own cell membrane, which suggests that they may trigger fratricidal killing by other CAR-T cells [40,41]. Moreover, Rui Mao et al. showed that CAR-T cells with moderate antigen binding affinity are not only less toxic, but also more effective in clinical response. They propose that a fast association and dissociation (high k_on_ and k_off_) kinetics of CAR-T-target engagement in a solid tumor allow CAR-T cells to generate sufficient signaling to kill tumor cells without being driven to exhaustion [42]. Anne Marijn Kramer tested, in her doctoral thesis, the binding of FCM63 and 4G7, cloned in an scFv-fc format, against CD19 via SPR Biacore kinetic screening and revealed KDs in a comparable range pointing FMC63 with the highest binding affinity (K_D_ = 0.881 nM) and 4G7 with a lower one (K_D_ = 20.1 nM). Moreover, in [39] it was shown that, besides FMC63, also, anti-CD19 CAR constructs, based on the scFvs of 4G7, elicit efficient effector function [39]. In contrast, we could not detect any specific binding of 4G7 antibody to our CD19-AD2 construct via BLI measurement. However, we were able to verify binding in an ELISA assay, but with exceptionally high concentrations of 4G7 (unpublished data). While FMC63 and 4G7 are conformationally sensitive epitopes and 3B10 recognizes a linear epitope, all three CD19 antibodies have partially overlapping, but distinct, epitopes [43]. Additionally, the HIB19 antibody was shown to bind the same or overlapping epitopes on CD19 compared to FMC63 [2,44].

## 4. Materials and Methods

### 4.1. Cell Culture

A recombinant CHO-K1 cell line, expressing the CD19-AD2 fusion protein, was used as a production factory (Lobner et al., 2020). The cultivation medium was CD-CHO (Thermo Fisher Scientific, Waltham, MA, USA), which was supplemented with 8 mM L-glutamine (Carl Roth, Karlsruhe, Baden-Württemberg, Germany), 0.5 mg/mL G418 (Thermo Fisher Scientific, USA), and anti-clumping agent (1:500 diluted, Thermo Fisher Scientific, USA) in TubeSpin bioreactor tubes (TPP Techno Plastic Products AG, Trasadingen, Schaffhausen, Switzerland) at 37 °C, 80% humidity, 7% CO_2_, and 220 rpm in a Kuhner shaker incubator. Cells were passaged twice a week routinely with a seeding density of 0.3 × 10^6^ cells/mL. Cell concentrations and viabilities were regularly monitored using a Vi-Cell XR cell counter (Beckman Coulter, Brea, CA, USA).

### 4.2. Bioprocessing

Bioprocess conditions were evaluated in 50 mL and 200 mL TubeSpin bioreactor tubes with 15 and 50 mL culture volumes. Semi-continuous perfusion conditions are realized by a complete medium exchange every 24 h by centrifugation at 300 G and 7 min. The temperature was reduced after reaching cell densities of 20 × 10^6^ cells/mL. For high cell density cultures and increased CD19-AD2 production, the CD-CHO cultivation medium was supplemented with either 1.65% or 3.3% Feed A + Feed B (Cytiva—Global Life Sciences Solutions Austria GmbH & Co KG, Linz, Upper Austria, Austria) in a ratio 10:1, when reaching at least 30 × 10^6^ cells/mL on day four. Semi-perfusion was performed for 11 days, and culture supernatants were collected according to the cultivation temperature.

### 4.3. Purification of CD19-AD2

Harvested supernatants from day 3 to day 11 were centrifuged at 300 g for 7 min and pooled, concentrated five times in case of 32 °C cultivation temperature and ten times when cultivated at 37 °C, as well as rebuffered in PBS with a 30 kDa TFF system (Pellicon XL-Ultrafiltrationsmodul, Biomax 100 kDa, 0.005 m^2^) and filtered (0.45 µM Supor R membrane filter, Pall Corporation, Port Washington, NY, USA).

For purification, the ÄKTA pure chromatography system (GE Healthcare, Chicago, IL, USA) was used. Concentrated, rebuffered, and diafiltrated supernatant was loaded onto a 5 mL HisTrap HP column (GE Healthcare, USA), and elution was performed by a stepwise gradient containing 25% and 45% of 500 mM imidazole solution in 20 mM phosphate buffer containing 150 mM NaCl (pH 7.4). Fractions were collected, and CD19-AD2 titers were determined with sandwich ELISA. In brief, the 96-well plate was coated with anti-HSA antibody (1:500 dilution; #A80-129A, Thermo Fisher Scientific, USA) and CD19-AD2 analyzed via biotinylated anti-human CD19 antibody HIB19 (1:500 dilution; #302204, Biolegend, San Diego, CA, USA) for 1 h at room temperature, followed by an incubation with streptavidin peroxidase (Roche Diagnostics GmbH, Mannheim, Karlsruhe, Germany) for 30 min and detected via Tecan Infinite M1000 PRO. The protein amount of pooled and purified CD19-AD2 was quantified via a NanoDrop Spectrophotometer, E1% (g/100 mL) = 17.27.

### 4.4. SDS-PAGE Analysis

The purification process of CD19-AD2 was monitored by using 4−12% Bis-Tris Protein Gels and NuPAGE MOPS SDS running buffer (all from Thermo Fisher Scientific, USA). Samples were mixed with NuPAGE LDS Sample buffer (4×) (Invitrogen, Waltham, MA, USA), heated to 70 °C for 10 min, separated on the gel, and Coomassie stained.

### 4.5. Binding Kinetics with Anti-CD19 Antibodies/Affinity Testing

Binding kinetics of CD19-AD2 to various commercially available biotinylated anti-CD19 antibodies, including FMC63 (Ab00613-2.0, Absolute Antibody Ltd., Oxford, Oxfordshire, UK), HIB19 (302204, BioLegend, USA), 4G7 (Ab00219-1.1, Absolute Antibody Ltd.), as well as 3B10 (TA506236AM, Origene Tech. Inc., Rockville, MA, USA), was measured by Bio-Layer Interferometry (BLI). All measurements were performed at 25 °C on an Octet RED96 instrument (Sartorius, Göttingen, Lower Saxony, Germany) and were recorded with the manufacturer’s software (Data Acquisition v11.1, Sartorius). Streptavidin (SA) biosensors (Octet^®^ SA Biosensors, Sartorius) were equilibrated in neutralization buffer (NB), containing PBS, 0.02% (*v*/*v*) Tween 20, and 1% BSA. The ligand samples (biotinylated anti-CD19 antibodies, as listed above) were diluted at 10 µg/mL concentrations. Analyte–sample dilutions of the purified CD19-AD2 construct were prepared from 10 to 600 nM concentrations in NB. Black 96-well plates (Nunc F96 MicroWell, Thermo Fisher Scientific, USA) were filled with 0.3 mL of each sample or buffer, maintained at 25 °C, and continuously agitated at 1000 rpm. Biosensors were equilibrated in NB for 10 min, and the baseline was recorded in the same buffer. The biosensors were then loaded with a ligand sample for 300 s, followed by an equilibration step in NB for 60 s. Association was monitored while dipping the functionalized biosensors in analyte solutions for 600 s or 1000 s. The dissociation was monitored by dipping the sensor into analysis buffer containing PBS. As controls, loaded and unloaded biosensors were measured without and with CD19-AD2 to see the systems’ variations or non-specific bindings. Kinetic data were processed with the manufacturer’s software (Data Analysis HT v11.1, Sartorius), and specific kinetic signals were fitted using a global fitting method, applying a 1:1 binding model.

### 4.6. Glycopeptide Analysis

CD19-AD2 was S-alkylated with iodoacetamide and digested with Trypsin or Chymotrypsin (Promega) or both. Glycopeptide analysis was performed by Liquid Chromatography-Electrospray Ionization–Mass Spectrometry (LC/ESI–MS/MS).

The digested samples were loaded on a nanoEase C18 column (nanoEase M/Z HSS T3 Column, 100 Å, 1.8 μm, 300 μm × 150 mm, Waters) using 0.1% formic acid as the aqueous solvent. A gradient from 1% B (B: 80% Acetonitrile, 0.1% FA) to 40% B in 45 min was applied, followed by a 10 min gradient from 40% B to 95% B, which facilitates elution of large peptides at a flow rate of 6 μL/min. Detection was performed with an Orbitap MS (Exploris 480, Thermo), which was equipped with the standard H-ESI source in positive ion, DDA mode (i.e., switching to MSMS mode for eluting peaks). Orbitrap resolution was set to 60,000, the scan Range was 350–3200, the RF lens was at 50%, the normalized AGC Target was 300%, and 3 microscans were performed. MS/MS scans included the following: an isolation window of 1.4 m/z, normalized collision energies of 25, 30, and 35, an Orbitrap resolution of 15,000, a normalized AGC target of 100%, 1 microscan, and a maximum injection time of 75 ms. MS scans were recorded (range: 350–3200 Da), and the eight highest peaks were selected for fragmentation. Instrument calibration was performed using Pierce FlexMix Calibration Solution (Thermo Fisher Scientific).

The possible glycopeptides were identified as a set of peaks consisting of the peptide moiety and the attached N-glycan varying in the number of HexNAc units, hexoses, fucose, and sialic acid residues. The theoretical masses of these glycopeptides were determined with a spreadsheet using the monoisotopic masses for amino acids and monosaccharides.

Manual glycopeptide searches were made using FreeStyle 1.8 (Thermo Fisher Scientific). For quantification of the different glycoforms, the peak intensities of the deconvoluted spectra were compared (annotation was performed using the in-house made software Glyco-parser (Freestyle_parser_v0.3.R; version 0.3)—github.com/lucaz88/Freestyle_parser accessed on 23 December 2022)). An online tool was used for the depiction of the found glycopeptide (https://glycotoolkit.com/Tools/GlycoGlyph/ accessed on 1 May 2023).

## 5. Conclusions

In this study, we have shown an optimized production and purification process under hypothermic conditions, leading to an increased yield of pure and stable CD19-AD2 fusion protein.

Of importance, we are the first to describe and identify the complex glycosylation patterns of each of the five N-glycosylation sites of human CD19, showing decreased high mannose-, terminal GlcNAc species, and increased terminal sialylation by hypothermic perfusion cultivations.

These findings will contribute to the further improvement and understanding of CD19-based CAR-T cell therapy approaches.

## Figures and Tables

**Table 1:** Kinetic binding constants of various immobilized anti-CD19 antibody ligands binding to the CD19-AD2 analyte in solution. Values are obtained after the global fitting of the binding signals obtained by BLI measurements. Signals detected above the level of non-specific binding were listed.

Analyte in Solution	Immobilized Ligand ID	k_on_ 10^5^/Ms	k_off_ [10^−4^/s]	KD (nM)
CD19-AD2	FMC63	2.06	6.57	3.19
	HIB19	0.44	16.99	38.29
	3B10	0.11	2.16	19.12
	4G7	no spec. binding		

## Data Availability

Data are contained within the article or supplementary material. The data presented in this study are available in the Supplementary Material and [13].

## Supplementary Materials

The supporting information can be downloaded at: https://www.mdpi.com/article/10.3390/ijms241310891/s1.
