# Supplementary material for: Recombinant Human CD19 in CHO-K1 Cells: Glycosylation Patterns as a Quality Attribute of High Yield Processes
Source: Int J Mol Sci. 2023 Jun 30;24(13):10891. doi: 10.3390/ijms241310891 (PMC10341778; doi:10.3390/ijms241310891)
Supplement: Supplementary file 1 [file ijms-24-10891-s001.zip › ijms-2440028-supplementary.pdf]

Supplementary Material

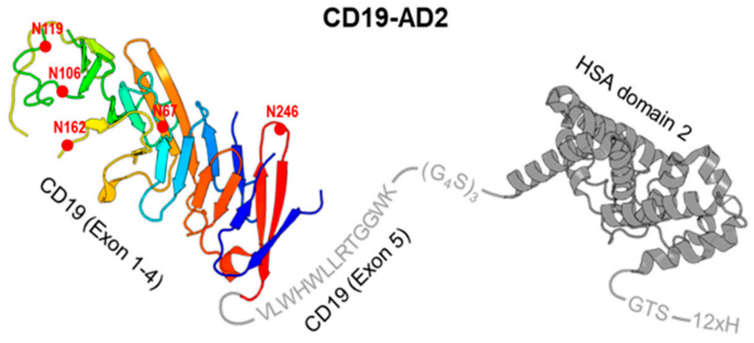

Supplementary Figure S1*: Schematic representation of CD19-AD2 fusion construct. The crystal structure of CD19 (PDB ID: 6AL5) encompassing exons 1–4 is depicted in rainbow colors from blue (N-terminus) to red (C-terminus) followed by exon 5 (displayed in light gray). This extracellular domain of CD19 is C-terminally fused via a glycine serine linker to domain 2 of human serum albumin (HSA; PDB ID: 6JE7), shown in dark gray. The fusion tag is followed by a polyhistidine tag. The five glycosylation sites (N67, N106, N119, N162, and N246) are indicated in red (as described in detail in Lobner et al., 2020).*

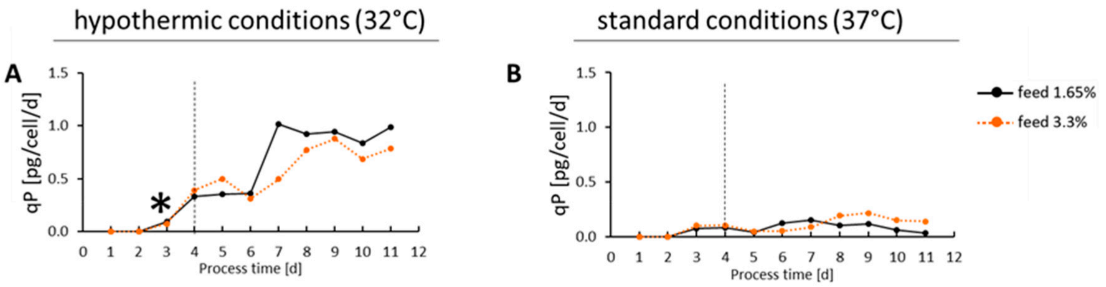

Supplementary Figure S2*: Cell specific production rate (qP) of CD19-AD2 from cells cultured (A) at 32°C vs (B) at 37°C. Temperature shift was performed on day three (marked with an asterisk). The start of feed supplementation is indicated by a dashed line at day four.*

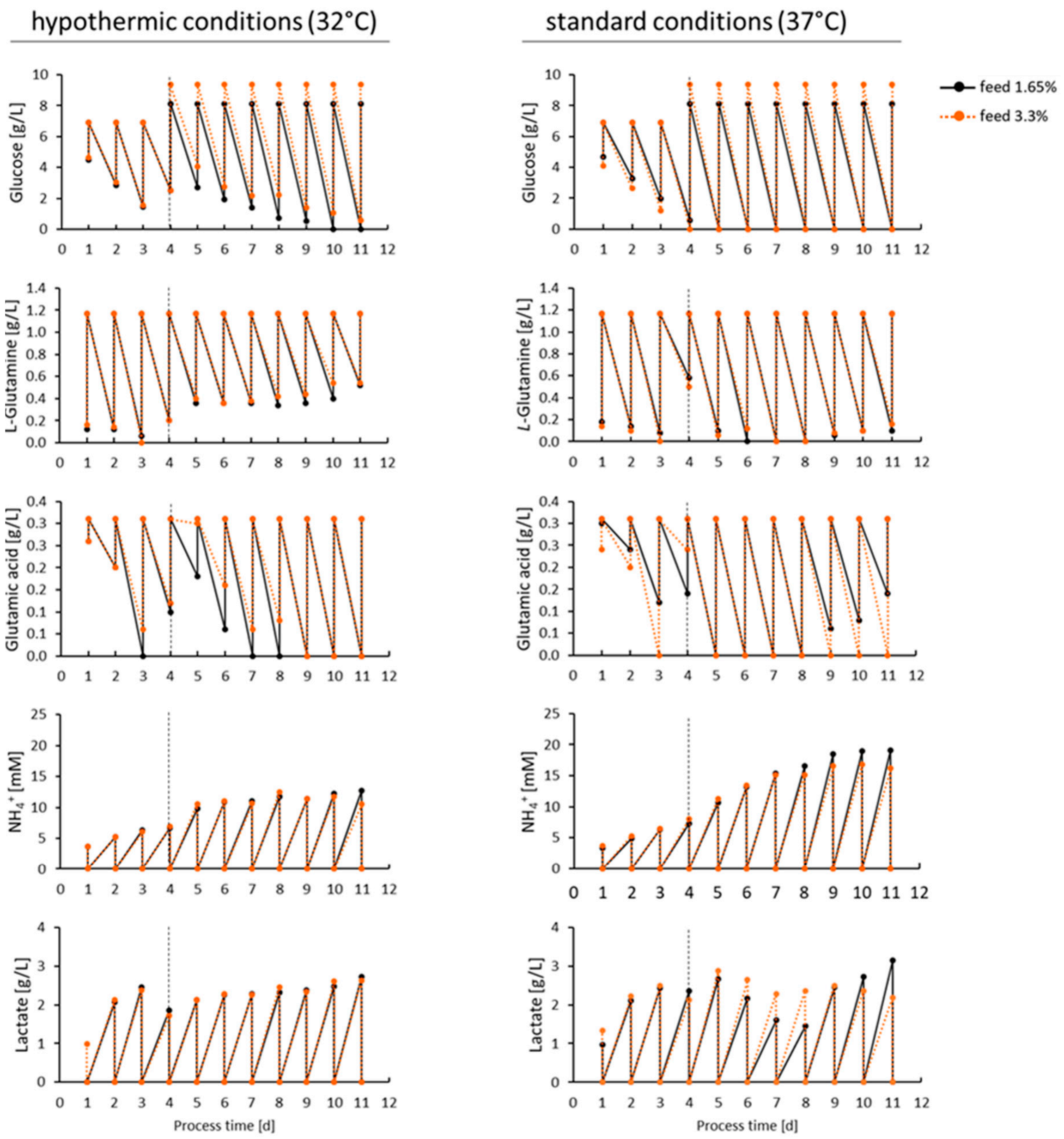

Supplementary Figure S3: *Analysis of metabolites during CD19-AD2 bioprocessing at 32°C (left panel) vs. 32°C (right panel) supplemented with 1.65% or 3.3% Feed A + Feed B in a ratio of 10:1. The temperature shift was performed on day three, start of feed supplementation at day four (indicated by a vertical, dashed line).*

A

Peptide: FNVSQQM

Mass: 966.4713 Da

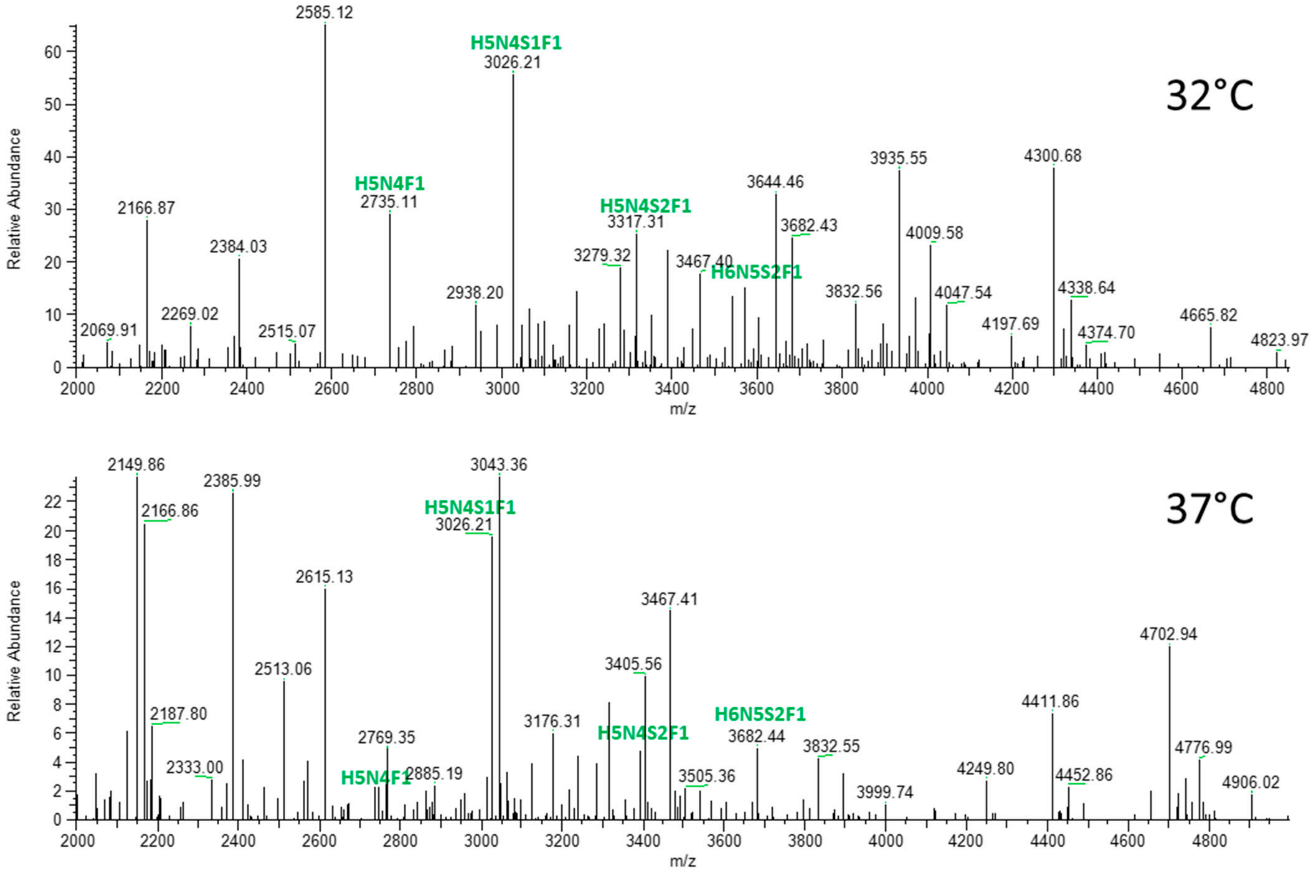

B Peptide: WNVSDLGGLGCGLK
Mass: 1475.7311 Da

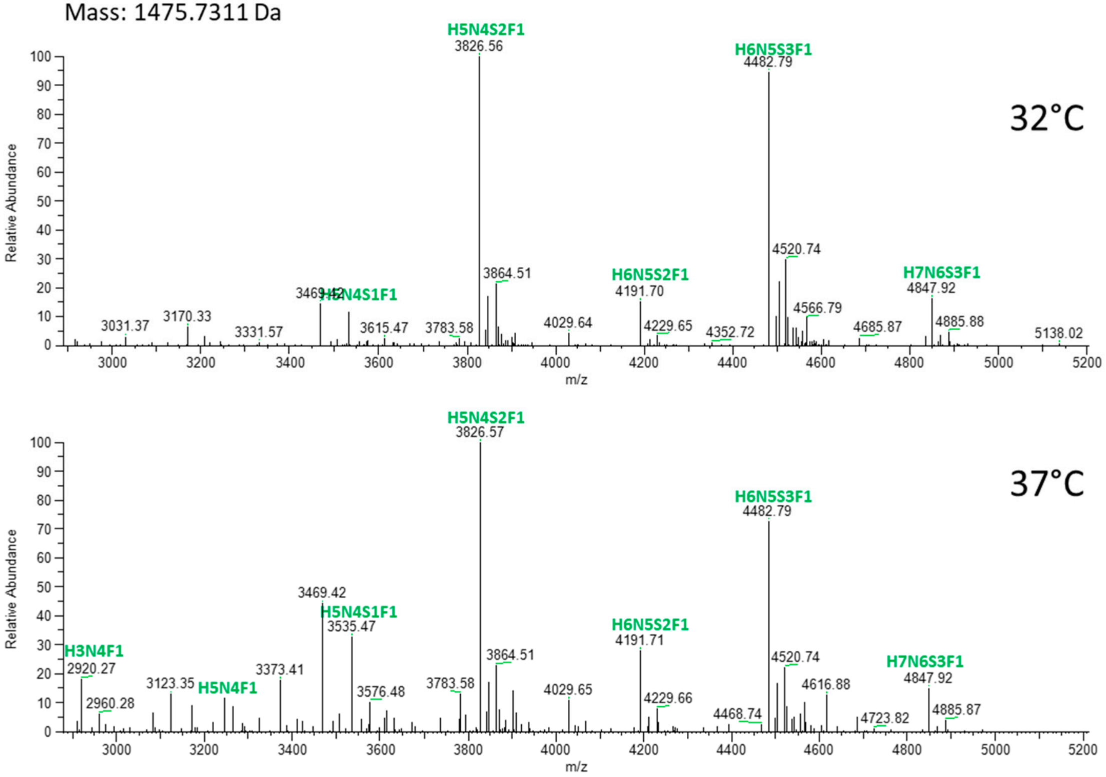

C

Peptide: GCGLKNRSSEGPSSPSGKLM

Mass: 2048.9851 Da

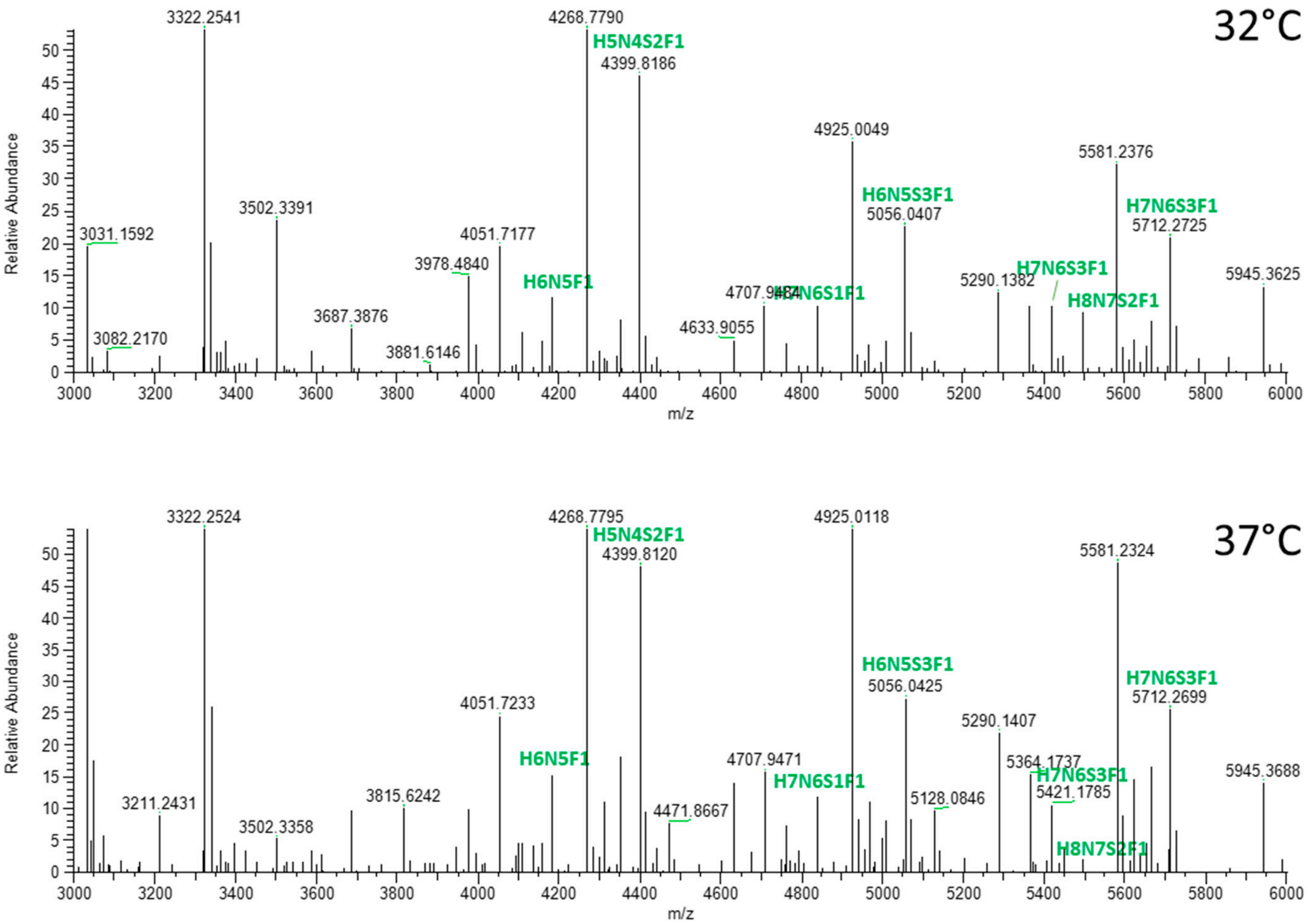

D Peptide: DSLNQSLSQDLTMAPGSTLWLSCGVPPDSVSR
Mass: 3418.6253 Da

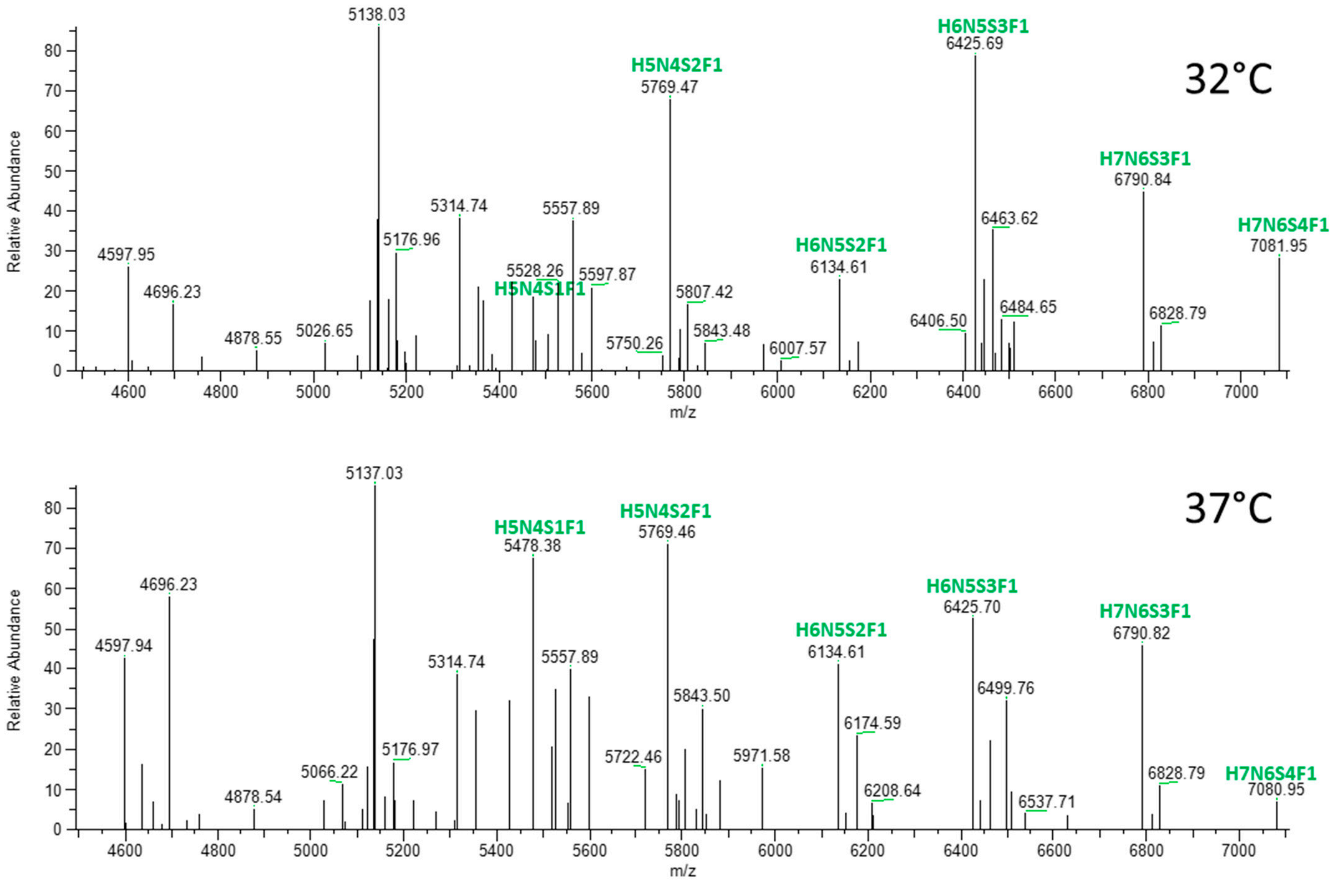

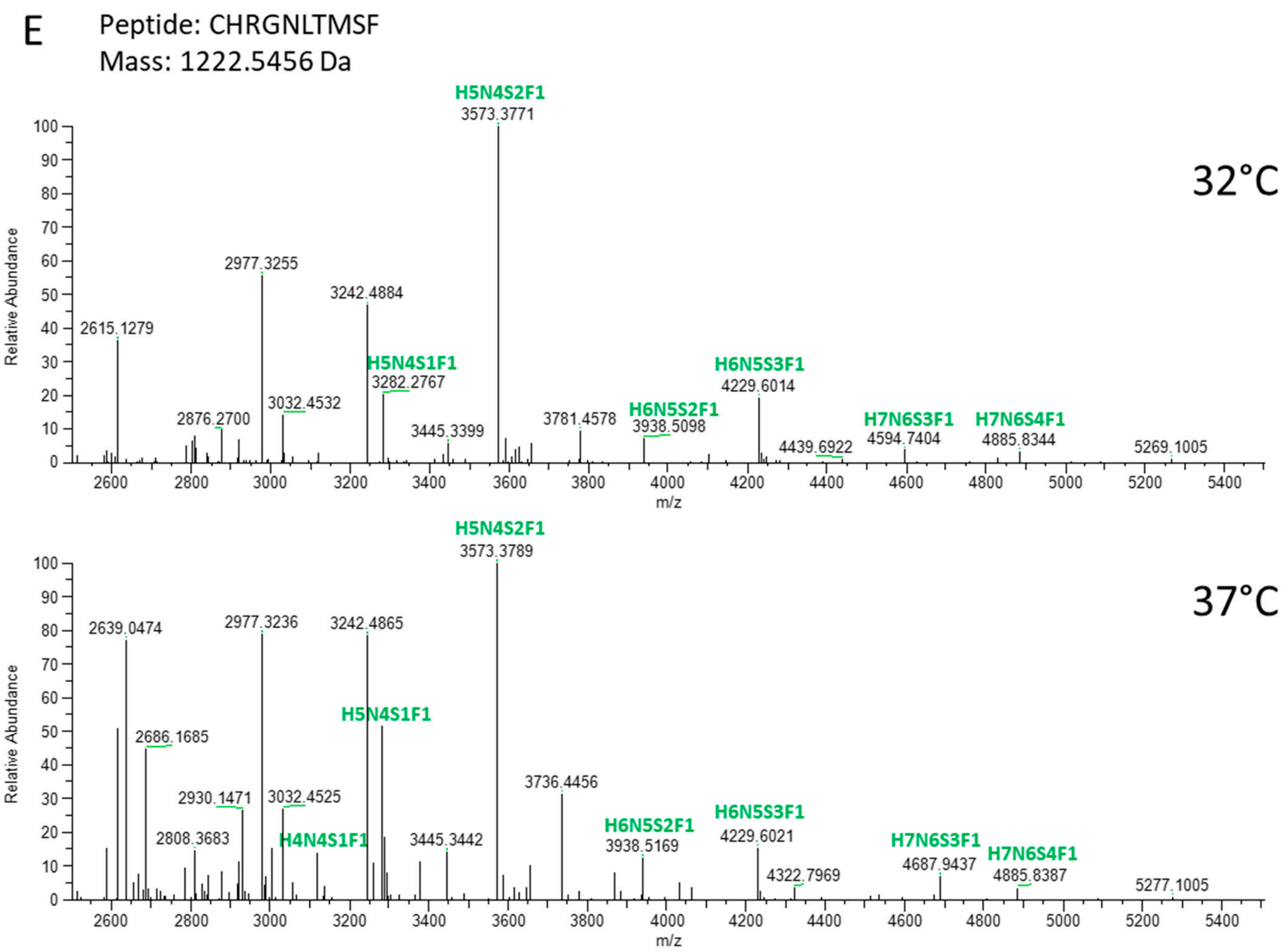

Supplementary Figure S4*: Mass spectra of the LC-ESI-MS/MS analyzed five N-glycosylation sites of CD19-AD2 produced at 32°C vs. 37°C. Glycosylation site: (A) N67, (B) N106 (C) N119 (D) N162 (E) N246.*

Supplementary Table S1*: LC-ESI-MS/MS analysis of the glycopeptides of the five potential N-glycosylation sites (N67, N106, N119, N162, N246). Relative proportions of the mainly complex type glycans of CD19-AD2 produced at 32°C vs. 37°C are listed (color-coded: non-glycosylated shown in gray, terminal sialylated (Sia) in purple, mannose-5 (Man5) in green, terminal galactose (Gal) in orange, terminal N-Acetylglucosamine (GlcNAc) in beige). Please note that only one possible isomer is presented since the used method only allows to determine the composition of the glycan.*

	Position									
	**N67**		**N106**		**N119**		**N162**		**N246**	
	IF**NVS**QQM		W**NVS**DLGGLGCGLK		GLK**NRS**SEGPSSPSGKLMSPKL		DSL**NQS**LSQDLTMAPGSTLWLS CGVPPDSVSR		DAGKYYCHRG**NLT**MSFHLEIT	
**Proglycan**	% 32°C	% 37°C	% 32°C	% 37°C	% 32°C	% 37°C	% 32°C	% 37°C	% 32°C	% 37°C
NaNa/4N5H2S		0.5	0.3	0.6						
NaNaNa/5N6H3S			0.3							
NaNaNaA/6N7H3S				0.1						
NaA	0.6	2.1								
NaAA/5N6H1S				0.2						
NaMan5(bi)/4N6H1S	0.9		0.1							0.6
NaNgF/5H4N1S1F1Ng			1.9	1.6	3.6	2.9	1.4	2.3	4.2	
NaNaA/5N6H2S			0.1	0.6						
NaNaF/4N5H1F2S	10.5	13.5	34.1	21.8	27.9	26.6	27.8	17.9	58.3	42.5
NaNaF(bi)/5N5H1F2S	0.6	1.1	1.5	2.5					0.6	1.3
NaMF/3N4H4FS		1.4	2.2	2.1				1.4	0.9	1.4
NaAF/4N5H1F1S	23.0	32.4	4.0	7.2		2.6		17.0	12.0	22.2
NaAF(bi)/5N5H1F1S	3.2	1.5	0.6	1.1						
NaGnF/4N4H1F1S	1.5	3.4	0.3	3.9	0.2	2.3		4.6	1.8	6.2
NaGnF(bi)/5N4H1F1S				2.3		0.5				
NaAAF/5N6H1F1S	9.3	7.6	1.1	3.2			3.0	7.6	0.7	1.9
NaAAAAF/7N8H1F1S					0.5	1.4				
NaNaAF/5N6H1F2S	10.2	8.2	5.2	6.1	2.8	4.5	9.4	10.3	4.3	5.4
NaNaNaF/5N6H1F3S	5.2		32.1	15.9	13.8	15.1	32.2	13.2	11.3	6.7
NaNaNaNaF/6N7H1F4S					12.7	14.3			2.1	1.6
NaAAAF/6N7H1F1S				0.5	6.2	5.7		1.7		
NaNaAAAF/7N8H1F2S				0.1	5.7	4.5				
NaNaAAF/6N7H1F2S			0.5	1.4			2.9			
NaNaNaAF/6N7H1F3S	5.0		5.7	3.4	6.4	6.0	18.3	11.5	2.3	
NaNaNaAAF/7N8H1F3S	0.7		0.6	0.3		0.9	1.9			
NaNaNaNaAAF/8N9H1F4S					2.0					
NaNaNaNaAF/7N8H1F4S					4.8					
NaNaNg/5N6H2S1Ng										
NaNgAF/5N6H1F1SNg1			0.4							
NaNgNgF/5N6H1F1SNg2			0.2	0.5						
NaNaNgAF/6N7H1F2S1Ng			0.3							
NaNaNg/5F6N1H2F1SNg			0.5	0.2	1.4					

Supplementary Table S1 continued on next page

AMF	2.6	4.2	0.2	0.8						
AGnF	1.3	6.6	0.2	1.5						2.2
AGnF(bi)				0.6						
AAF	12.1	3.9	0.5	2.6		0.3			0.4	3.1
6N5H2F	3.1									
AAF(bi)	4.9	1.4		0.5						
AAAF/5N6H1F	3.7	2.4	0.2	1.0	7.2	8.2		1.7		
AAAAF/6N7H1F					0.4					
NgAAAF/6N7H1FNg1					0.6	0.5				
MGnF		2.6	0.3	1.9				1.8		1.6
GnGnF		6.9	0.5	4.0		0.5				3.4
GnGnF(bi)/N5H3F			0.4	2.9		0.2		2.9		
NgNgF/5H4NF2Ng						0.8				
Man4			0.1	0.4						
Man5			0.7	3.9				4.2		
Man5Gn	1.7									
Man5Gn(bi)		0.4								
Man7			0.2	0.4						
Man8				0.4						
MMF				0.4						
MM/3H2N				0.3						
non-glycosylated peptide			1.1	1.8						
**Terminal Sia [%]**	70.7	71.7	95.8	76.4	91.8	89.5	100.0	89.5	99.6	89.7
**Terminal Gal [%]**	27.6	18.5	1.1	7.1	8.2	9.0	0.0	1.7	0.4	5.3
**Terminal GlcNAc [%]**	0.0	9.5	1.2	8.9	0.0	1.5	0.0	4.7	0.0	5.0
**Man5 [%]**	1.7	0.4	0.9	5.8	0.0	0.0	0.0	4.2	0.0	0.0
**non-glycosylated [%]**	0.0	0.0	1.1	1.8	0.0	0.0	0.0	0.0	0.0	0.0
**sum**	100.0	100.0	100.0	100.0	100.0	100.0	100.0	100.0	100.0	100.0
